# Bacterial Cellulose Production, Characterization, and Its Use as a Membrane Filter for Microorganism

**DOI:** 10.1021/acsomega.6c01921

**Published:** 2026-08-19

**Authors:** Bahar Samyeli, Filiz Boran, Ozfer Yesilada, Suleyman Koytepe

**Affiliations:** † Biology Department, Arts and Science Faculty, 37520 Inonu University, Malatya 44280, Turkey; ‡ Chemistry Department, Arts and Science Faculty, Inonu University, Malatya 44280, Turkey

## Abstract

The aim of this study was to investigate the usability of bacterial cellulose (BC) produced by *Gluconacetobacter xylinus* NRRL B-759 (*Komagataeibacter xylinus*) as a membrane filter and to determine the shortest incubation time for obtaining the BC membrane with an effective removal capacity. BC production of this bacterium was found to be dependent on incubation time (day), and the properties of the membranes produced on each day were then examined in detail. BC formed on the third day showed a suitable peak structure and ideal formation of the cellulose structure, as determined by FTIR and X-ray analyses, respectively. SEM analysis also confirmed that the membrane structure obtained on the third day was optimal. All membranes produced from the second day onward exhibited sufficient mechanical properties. TGA and DSC analyses showed that the BC membrane structures were thermally stable at approximately 200 °C and exhibited suitable flexibility at 20 °C. To evaluate the potential of BC membranes as filters, the prepared *Escherichia coli* and *Staphylococcus aureus* suspensions were filtered through the third-day BC membranes under sterile conditions. No bacteria were detected in the filtrate, indicating the effective microorganism retention. Reusability tests revealed that these membranes could be repeatedly used in the filtration process. SEM, EDX analyses, and surface elemental mapping of the membranes used for filtration of *E. coli* and *S. aureus* showed that the bacterial cells on the membrane surface and unlike the pure membranes (the membranes without filtration) exhibited the presence of nitrogen, phosphorus, and sulfur peaks. The results of this study demonstrated that BC membranes could be used effectively in microorganism-filtration applications. The BC membrane may also serve as an effective biologic material for filtration of various pollutants.

## Graphical abstract

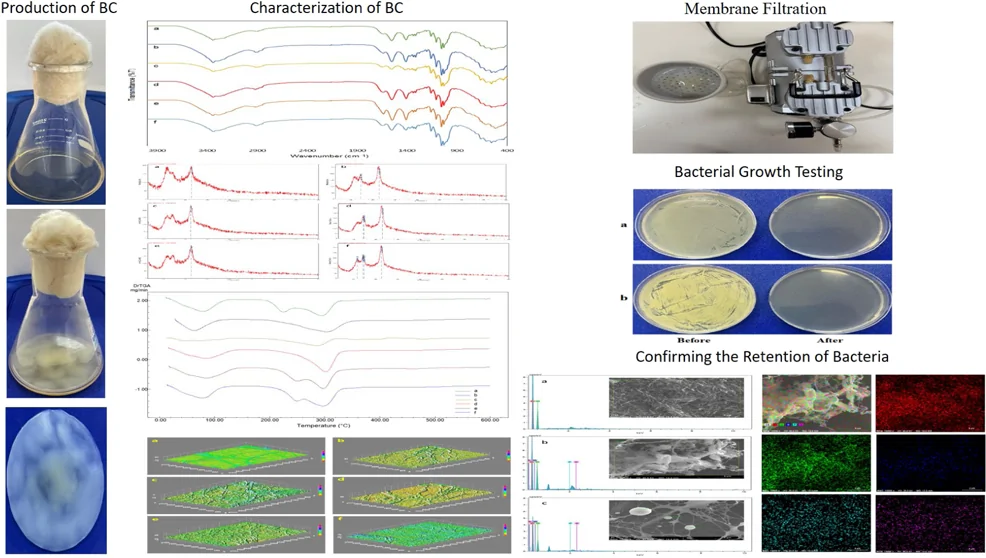

## 1 Introduction

Waterborne pathogen contamination and associated diseases remain as major water quality concerns worldwide.^1^ The discharge of untreated or insufficient wastewater leads to the pollution of drinking-water resources. At the same time, new pollutants that have not been regulated in terms of processing or treatment also pose a significant problem.^2^ Consequently, the removal of pollutants from water resources and wastewater is a critical issue.^3^ As water pollution has reached alarming levels, the demand for high-quality water continues to increase.^4^ Water contamination poses a significant problem, especially in developing countries, where hundreds of millions of people have difficulty accessing safe drinking water.^5^ According to the World Health Organization, approximately two billion people consume water contaminated with feces and more than 500,000 deaths per year are attributed to diarrheal diseases caused by unsafe water.^6^

Membrane filtration is an effective physical method for removing microorganisms from the water and wastewater. This method is based on the retention of substance or microorganism on a membrane surface and enables the separation of substances and microorganisms according to their sizes.^7^ Currently used membranes are generally composed of polymeric or inorganic (ceramic) materials. Common polymeric membranes include polysulfone, poly­(ether sulfone), polyvinylidenefluoride, polyacrylonitrile, and cellulose acetate. Different membranes can be used in applications ranging from microfiltration to reverse osmosis.^8^ However, most common water treatment technologies rely on synthetic materials such as ion-exchange resins or polymer membranes/filters, whose synthetic nature is a disadvantage. Ceramic membranes, which are also widely used, require a high energy input.^2^

Even when the initial water source is clean, the quality of water deteriorates during storage. Membrane filtration offers the advantage of physically removing microorganisms without the need for chlorination, thereby avoiding the formation of harmful products.^9^ Additional advantages of membrane applications include their simple operation and no chemical use. Since synthetic membranes cannot be biologically degraded, they create a waste problem, and harmful chemicals can be used during their synthesis.^3,9^ As a result, there is a growing interest in biobased membrane filters. Materials that are biodegradable, environmentally friendly, and cost-effective are crucial for water treatment technologies.^2^

Cellulose is the most abundant biopolymer in the world and is primarily derived from plant cell walls. However, plant celluloses are not in pure form and are typically associated with lignin, pectin, and hemicellulose. Therefore, plant-based celluloses must first be purified in order to be used in applications. Some bacterial species and algae can also produce cellulose.^10^ Bacterial cellulose (BC) is a natural nanocellulose polymer synthesized by some bacteria. *Gluconactobacter xylinus* (*Acetobacter xylinum*) is the most efficient BC producer. This is a linear biopolymer,^11^ and unlike plant cellulose, BC is produced in a highly pure form free of lignin, hemicellulose, and pectin. They have many important unique properties. Especially its structural, physicochemical, and mechanical properties are superior to plant cellulose.^10^ Therefore, its purification is easier and requires low energy compared to plant-derived celluloses.^12^

BC exhibits superior physicochemical properties and mechanical properties including high crystallinity, excellent tensile strength, thermal stability, ultrafine fiber network structure, hydrophilicity, nontoxicity, transparency, and moldability.^13^ These properties make BC an attractive material for various environmental applications. The high cost of commercially available filtration membranes has prompted an increase in research into the development of sustainable and environmentally friendly alternatives. Bacterial cellulose has high purity, excellent mechanical strength, and particularly an environmentally friendly nature. Therefore, their use as a membrane filter could be advantageous.

In particular, due to its environmentally friendly nature, BC is a promising, sustainable alternative to conventional polymer-based membranes for water filtration.^14^ BC biomembranes provide better performance for application in various industries and are candidates to replace widely used synthetic materials. High porosity and reticulated structure with small diameter pores (less than 100 nm) make biomembranes ideal for higher-quality filtration.^15^ BC formed as a layer on the surface of the medium has been proven to have high tensile strength, elasticity, flexibility, durability, shape retention, and high water retention capacity and is also nontoxic and nonallergenic. Its high water retention capacity makes it suitable for the filtration of colloids or particles in the micro- to ultrarange.^16^

Recent studies have studied BC-based materials as membrane filters for water treatment applications including for the removal of pathogens, wastewater contaminants, heavy metals, and pollutants.^17,18^ Alves et al. (2020) reported that BC membranes effectively removed *Escherichia coli* from natural water samples collected from the Igarassu River basin in Pernambuco, Brazil.^6^ Similarly, BC produced by *Komagataeibacter nataicola* TISTR efficiently removed bacteria.^19^ According to our literature knowledge, there are limited studies on the use of BC as a membrane filter for bacterial removal. Therefore, the BC production capacity of *Gluconacetobacter xylinus* NRRL B-759 was investigated as a function of incubation time (0–7 days) to determine the shortest incubation day required to obtain the BC membrane with effective microorganism removal capacity. Subsequently, BC membranes obtained at different incubation periods were characterized, and the usability of bacterial cellulose as a membrane filter for the removal of microorganisms was evaluated.

## 2 Materials and Methods

### 2.1 The Bacterium Used for Bacterial Cellulose Production

In the study, *Gluconacetobacter xylinus* NRRL B-759 strain was used for bacterial cellulose (BC) production. This bacterium was provided by Dr. Aynur Gül KARAHAN and is stored as a pure culture in the Biotechnology Laboratory of Inonu University Biology Department, Malatya, Turkey. This bacterium is transferred onto a sterile fresh Hestrin Schramm (HS) agar medium every 4 weeks, incubated statically at 30 °C for 48 h, and then stored in the refrigerator at +4 °C. The sterile HS agar medium (g/L) contains glucose 20, peptone 5, yeast extract 5, Na_2_HPO_4_ 2.7, citric acid 1.15, and agar 15.

### 2.2 Preparation of Stock Culture Used in Bacterial Cellulose Production

The sterile fresh HS liquid medium without agar was used for the production of stock bacterial culture. First, 50 mL of HS liquid medium/250 mL of Erlenmeyer flask was sterilized by autoclaving at 121 °C for 20 min. After sterilization, *G. xylinus* stock solid culture was inoculated into this liquid medium and the culture was incubated statically at 30 °C for 10 days. Then, 1 mL of this culture was inoculated into a sterile 50 mL HS liquid medium/100 mL Erlenmeyer flask. This culture was incubated statically at 30 °C for 10 days, and this bacterial culture was used as a stock liquid bacterial culture.

### 2.3 Production and Obtaining the Bacterial Cellulose Membranes

1 mL of the stock bacterial culture was inoculated into 250 mL of Erlenmeyer flasks containing 40 mL of sterile HS liquid media, and the cultures were incubated statically at 30 °C for 7 days. These cultures were filtered on the second, third, fourth, fifth, sixth, and seventh days, and therefore, the BC membranes produced on these days were obtained. The obtained BCs were washed with distilled water several times and then, they were purified. In order to purify, these membranes were placed in a 0.1 M NaOH solution and kept in a water bath at 75 °C for 2 h. After that, the membranes were washed with distilled water several times. As a result, pure wet form BC membranes were obtained. Then, some of these membranes were kept in wet form and some of them were lyophilized.^20^

### 2.4 Characterization of Bacterial Cellulose Membranes

An elemental analysis technique was used to perform the elemental structures of the membranes. The elemental contents per unit were compared theoretically and experimentally. The chemical properties of the time-dependent bacterial cellulose structures were confirmed by FTIR analysis. FTIR analyses were performed in the range of 400–4000 cm^–1^ with a PerkinElmer two-model FTIR spectrophotometer. All FTIR analyses of the prepared bacterial celluloses were performed with a 4 cm^–1^ measurement sensitivity using the ATR measurement mode.

The crystallinity level of bacterial cellulose structures is particularly important in terms of the mechanical stability of the membrane structure. Therefore, the crystallinity level of bacterial cellulose structures was determined by X-ray spectrophotometer analysis. Rigaku Geigerflex D/Max B model X-ray spectrophotometer was used in these analyses. All X-ray spectrometric analyses were carried out in the 2°–80° theta measurement range with a measurement sensitivity of 0.2° theta. Membranes used for these analyses were cut into 1 cm × 1 cm dimensions, and X-ray analyses were carried out.

The most fundamental characteristics of a membrane structure are the membrane pore structure, pore morphology, and pore size. Detailed SEM analyses were used to examine these characteristics and the surface morphologies of the prepared membranes. The SEM measurements were performed using an SEM LEO 40 model SEM. Before SEM analysis, all samples were coated with a 20 nm thick gold palladium using a BAL-TEC SCD 050 brand sputter. Fiber diameter, void distribution, and surface roughness of bacterial cellulose structures were determined. SEM measurements were performed at different magnifications. Surface roughness measurements were taken from the 500× and 1000× images obtained as a result of the measurement using the ImageJ program.

During the BC production process, significant changes can be observed in the thermal properties of the produced cellulose as time progresses. This is entirely related to the stacking of cellulose chains over time and the increasing interactions between them. An increase in thermal properties occurs depending on the secondary interactions of the cellulose chains during production and the chain stacking density value. To determine these changes, TGA and DSC analyses were performed on bacterial cellulose produced over time. Thermal stability and glass transition temperatures of the obtained bacterial cellulose membranes were examined by TGA/DSC. In TGA measurements, samples were analyzed at room temperature with a heating rate of 10 °C/min up to 600 °C. Each sample was examined with a Shimadzu TGA 50 device by heating in air atmosphere. The glass transition temperatures (*T*_g_) of the obtained BCs were determined using a Shimadzu DSC 60. A 5 mg sample was used, and the samples were heated to 300 °C at room temperature in a static air atmosphere by applying a heating rate of 10 °C/min. All DSC analyses were analyzed with a 5 mg α-alumina reference material.

The mechanical strength of cellulose largely depends on the hydrogen bonds between polymer chain groups. As the production time increases, the molecular weight and average degree of polymerization can increase. These changes improve the thermal, structural, mechanical, and morphological stability of the polymer over time. Therefore, the changes in the mechanical properties of structures obtained over time during the bacterial cellulose production process were investigated. The mechanical properties of the membranes were determined with the MT mechanical test analyzer. Each membrane was cut into thin strips of 1 cm × 12 cm. One cm × 10 cm measurement gap was created and subjected to mechanical tensile testing. The tensile process was carried out at room temperature and approximately 60% relative humidity. Each sample was measured three times, and the results were given as the mean ± standard deviation. The mechanical analysis of each membrane was carried out for both the wet and lyophilized forms.

### 2.5 Growing the Bacteria for Membrane Filtration Process

Gram-negative *E. coli* and Gram-positive *Staphylococcus aureus* bacteria were used to test the applicability of bacterial cellulose as a membrane filter. They were grown on Mueller Hinton agar (MHA) media. For this purpose, the prepared MHA medium was autoclaved at 121 °C for 20 min and then poured into Petri dishes. Then, solid cultures of *E. coli* and *S. aureus* were separately inoculated onto these MHA media and the cultures were incubated statically at 37 °C for 24 h. After incubation, solid bacterial cultures were taken with a culture stick and 0.5 McFarland microbial suspensions were prepared separately. Then, this suspension was diluted to a final inoculum value of 1.5 × 10^6^ CFU/mL and they were used in the membrane filtration process.

### 2.6 Use of Bacterial Membranes as Membrane Filter

The characterization studies showed that the membranes obtained on the third day were suitable for use as membrane filters, and therefore, these membranes were selected for use as filter. 10 mL of the microbial suspensions prepared as stated above were separately filtered, using a vacuum pump, from the wet BC membranes placed in Buchner funnels under sterile conditions. A 10 mL of the microbial solution was filtered through a membrane with a diameter of 80 mm. After filtration, 1 mL was taken from each filtrate under sterile conditions and serial dilutions were performed. Then, 0.1 mL from these diluted samples was placed and spread on the surface of MHA media. These cultures were incubated statically at 37 °C for 24 h. After incubation, the number of colonies in MHA media was counted and CFU/mL was calculated. The unfiltered bacterial suspensions were also used as control groups. The consecutive five cycles of the filtration were also employed to evaluate the reusability of the membrane for filtration. For this purpose, after the bacterial solution had been filtered through the membrane, a subsequent bacterial solution was filtered through the same membrane.

The usability of BC for the filtration of textile dye Astrazon Red dye and model dye Methylene Blue, as well as waste oil/water mixture was also investigated. Dye solutions were prepared at a concentration of 10 mg/L and then filtered from BC. The absorbance of Astrazon Red and Methylene Blue dyes was measured spectrophotometrically at 530 and 663 nm, respectively. For filtration of waste oil/water mixture, a mixture consisting of 10 mL of waste oil and 10 mL of water was prepared and filtered through BC. The viscosity of the filtrate was measured at 25 °C using a Brookfield CAP 2000+ Viscometer. Measurements were performed with a sample volume of 25 μL and eight replicates. Each measurement was performed using 25 runs at 20 rpm.

## 3 Results and Discussion

The production and applications of bacterial cellulose (BC) have attracted considerable attention from researchers.^20,21^ One promising application of BC is its use as a membrane for filtration of bacteria. In this study, the BC production capability of *G. xylinus* in HS medium was first evaluated as a function of the incubation time to determine the shortest day required to produce BC membrane, thereby obtaining an environmentally friendly membrane.

Subsequently, the synthesized BC membranes were characterized in terms of their structural, morphological, thermal, and mechanical properties. Finally, the usability of BC as a membrane for the filtration of bacteria was evaluated.

### 3.1 Characterization of Bacterial Cellulose

The bacterial cellulose samples were first examined by elemental analysis. The theoretical elemental yield of BC, consisting of C, H, and O, showed good agreement (98.6%) with experimental results. The theoretical values and experimental parameters obtained as a result of these analyses are presented in Table 1. The theoretical elemental analysis results confirmed the experimental data, particularly from the second onward. However, on later days, especially the sixth and seventh days, the theoretical agreement decreased to approximately 96%. This reduction is attributed to the accumulation of metabolites and byproducts of bacterial metabolism as well as an increased amount of bacterial cells, which caused a partial deviation in the elemental analysis results. The elemental analysis results indicated that the optimum BC structure was obtained on the third day. The strong agreement between the theoretical and experimental C and H values confirmed the formation of BC with the desired structural properties.

**Table 1:** Elemental Analysis Results of the BCs

day		C	H
2	calculated	58.19	4.85
	found	53.30	7.96
3	calculated	58.19	4.85
	found	58.04	4.59
4	calculated	58.19	4.85
	found	57.11	4.62
5	calculated	58.19	4.85
	found	58.67	4.69
6	calculated	58.19	4.85
	found	58.72	5.04
7	calculated	58.19	4.85
	found	59.21	5.26

FTIR analysis was performed to identify the functional groups of the BCs. The FTIR spectra of BCs obtained on the second, third, fourth, fifth, sixth, and seventh days are shown in Figure 1. No significant differences were observed among the spectra recorded on different days. In particular, the spectra obtained on the second and third days revealed a clear spectrum that is consistent with those reported in the literature. A broad absorption band in the range of 3000–3600 cm^–1^ was clearly observed, corresponding to the H bond stretching vibration of the surface hydroxyl groups in the cellulose structure.^22,23^ Additionally, an O–H stretching peak appeared around 1600 cm^–1^ originating from hydroxyl groups. The aliphatic C–H stretching vibration peaks of the cellulose backbone were observed in the spectral range of 2800–2950 cm^–1^.^6,22,23^ The C–C stretching vibrations at 1420 cm^–1^ and stretching vibrations belonging to –CH_2_– units were determined at 1500 cm^–1^. The C–O stretching vibrations appeared at around 1061, 1112, and 1164 cm^–1^ due to the C–O structure.^23^ These peaks were C–O–C etheric stretching vibrations belonging to the calcic cellulose structure, and in addition, a low-intensity stretching vibration peak was observed at 928 cm^–1^ due to the 1-4-β-glycoside bond between the glycoside units. The presence of all these peaks indicated that the desired BS structure had begun to form by the second day. However, the peak structure and intensity observed on the third day were clearer and more distinct with increased sharpness and intensity of the peaks. This suggests that a more suitable BC structure was obtained on the third day. In contrast, the BC formed on the first day was very thin and mechanically weak, lacking membrane integrity. Therefore, infrared analysis was not performed for the first-day sample. As the membrane structure and mechanical durability improved in the subsequent days, infrared analyses were performed. Prior to analysis, all samples were washed with pure water; therefore, no impurity-related peaks were observed in samples obtained on the fifth, sixth, and seventh days. Only the characteristic peaks corresponding to the pure cellulose structure were clearly detected.

**Figure 1:**
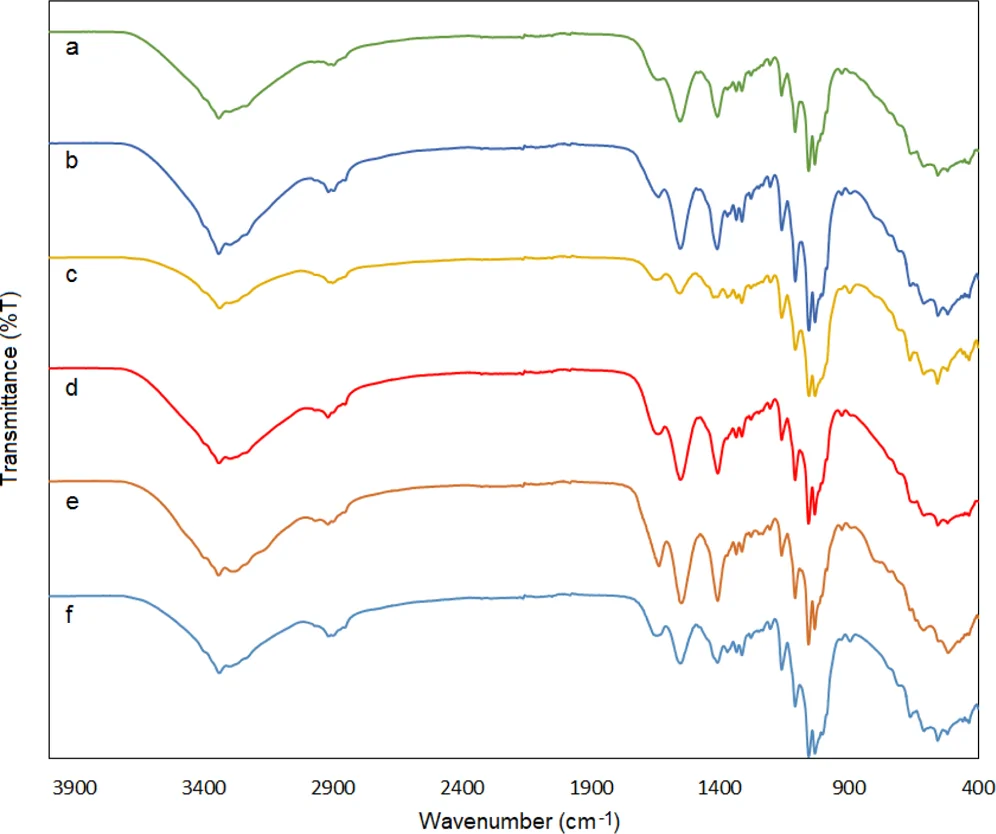
FTIR spectra of BC samples obtained at different incubation times: (a) second day, (b) third day, (c) fourth day, (d) fifth day, (e) sixth day, and (f) seventh day.

The crystallinity of BC is an important parameter for its applications. The stability and regular structure of polymers with long molecular chains depend on the interactions between the chains, chain stacking density, and chain polarity. In cellulose, a single chain unit can form up to three H-bonds with the other chains, resulting in very strong interaction between the chains. Because of these strong hydrogen bonds, cellulose units do not easily dissolve or melt. They have high stability, and as cellulose chains stack, they form crystalline layers. The hydrogen bonds, particularly those present in the cellulose molecule, are generally disordered and are randomly distributed. The rearrangement of one or more of these bonds can lead to the formation of different crystalline cellulose structures. The crystalline structure of cellulose was first observed in 1914 using X-ray diffraction.^24,25^ Since then, various theories have been proposed, with the structure introduced by Meyer and Misch being the most prominent.^26,27^ In this structure, the native, unprocessed form of cellulose is termed cellulose-I, which can undergo changes depending on factors such as chemical treatments and temperature.

Some special cellulose forms such as cellulose II in extremely alkaline conditions, cellulose III in mildly alkaline conditions, and cellulose IV at high temperatures such as 280 °C have been well characterized under specific conditions. In this study, XRD analyses were performed to investigate the crystalline forms of the BCs and to determine their degree of crystallinity. The resulting X-ray spectra are presented comparatively in Figure 2. Examination of these spectra revealed two main diffraction peaks, observed particularly approximately 12° and 22° theta.^23^ Starting from the third day, a third peak was detected around 12° theta. The peak around 22° theta corresponds to the classical (200) peak of the cellulose structure.^28^ Additionally, peaks at 1.01 around 14° theta and 1.10 around 15° theta are clearly visible in the cellulose structure. Examination of the X-ray spectra indicated that the targeted cellulose structure was well formed, particularly on the third day. Furthermore, after the fourth day, the peak at 14.5° theta became more pronounced, suggesting that the cellulose structure stacks on top of each other. These observations confirm that the desired and optimum BC structure was formed by the third day.

**Figure 2:**
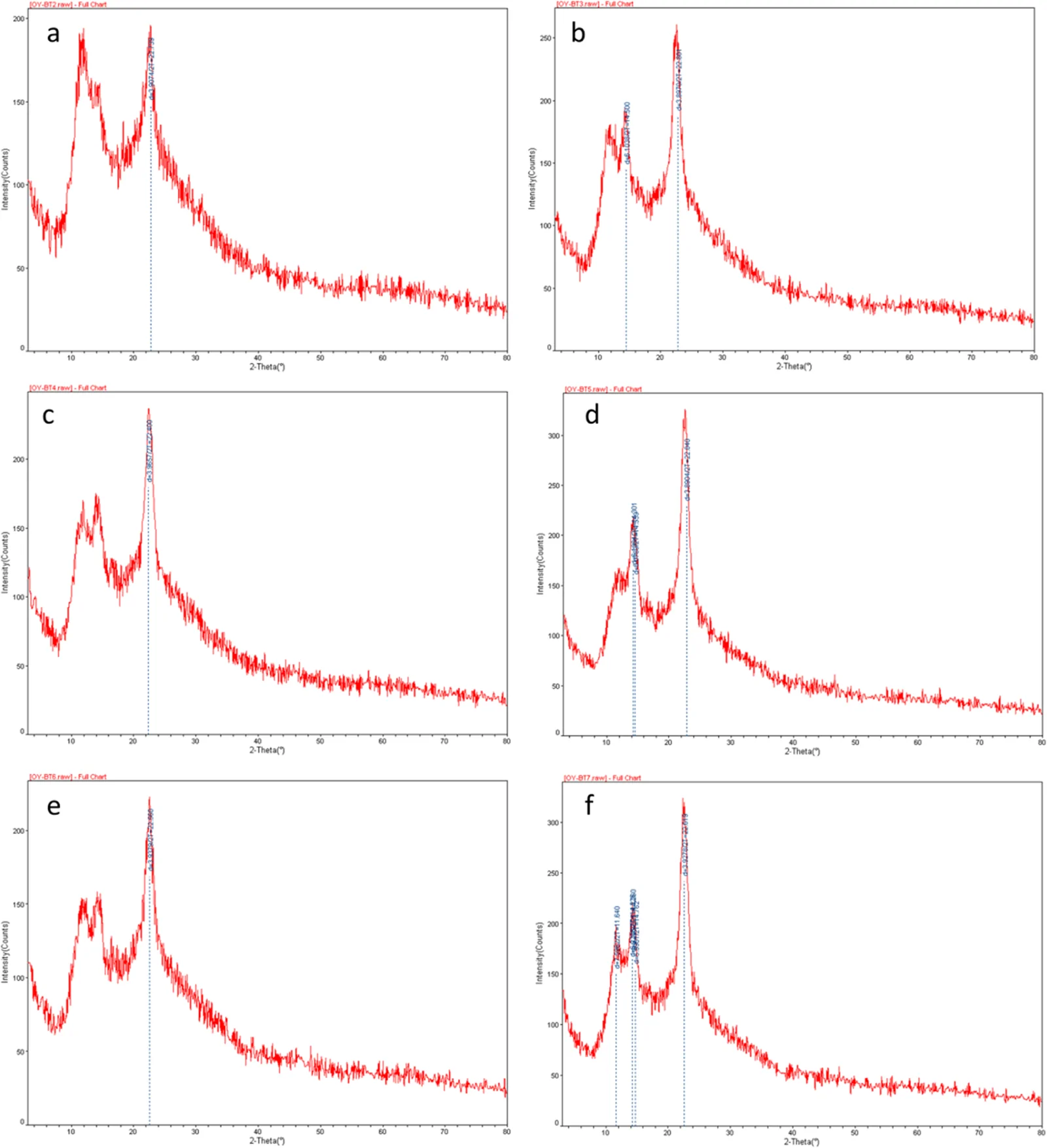
X-ray spectra of BC samples obtained at different incubation times: (a) second day, (b) third day, (c) fourth day, (d) fifth day, (e) sixth day, and (f) seventh day.

Using these spectrum values, the crystallinity index (C.I.) (%) was calculated according to the following formula^29^

$$\%\;C.I.=\frac{I_{200}-I_{amorphous}}{I_{200}}\times 100$$

In this formula, the *I*_200_ peak corresponds to the (200) crystalline plane and is observed around 22° theta, while the *I*_amorphous_ value represents the intensity of the amorphous structure around 12°. The percentage crystallinity values of the BC structures are given in Table 2. The crystallinity values recorded from day 2 to day 7 were 66.55, 72.40, 69.02, 75.69, 69.51, and 74.69, respectively^23^. The results indicate a significant crystallinity of approximately 70% on day 3, demonstrating the BC with the desired crystallinity ratio on day 3.

**Table 2:** Crystallinity Index (%) Values of BCs from Day 2 to Day 7

day	crystallinity index (%)
2	66.55
3	72.40
4	69.02
5	75.69
6	69.51
7	74.69

The BCs obtained in this study exhibit particularly high potential as a biomaterial.

Thermogravimetric analyses (TGA) were performed to confirm the presence of stable cellulose structures capable of withstanding high-temperature treatments. These analyses provided information on moisture content, degradation onset temperature, thermal stability, and char yield. The TGA results are presented in Figure 3A, while the differential thermogravimetric analysis results are given in Figure 3B. As shown in Figure 3A,B, three main weight losses were observed. The first weight loss, occurring between 80 and 120 °C, is due to the removal of moisture from the BC. The most significant weight loss observed between 260 and 320 °C is due to the cleavage of the C–O–C bonds within the cellulose structure. At temperatures above 400 °C, further weight loss occurred due to the thermal degradation of cellulose, resulting in the formation of char. The main degradation temperature was determined to be around 280 °C, while the onset was around 270 °C.^22^ This result showed that the BC samples are thermally stable up to this temperature.^22,23^ Residual weight analysis revealed a residue of about 10%, consistent with the high purity of the BC. The DTG analysis further clarified the weight loss. The structural stability and thermal resistance were observed to improve from day 3 onward, corroborating the XRD results and confirming the development of thermally stable BCs.

**Figure 3:**
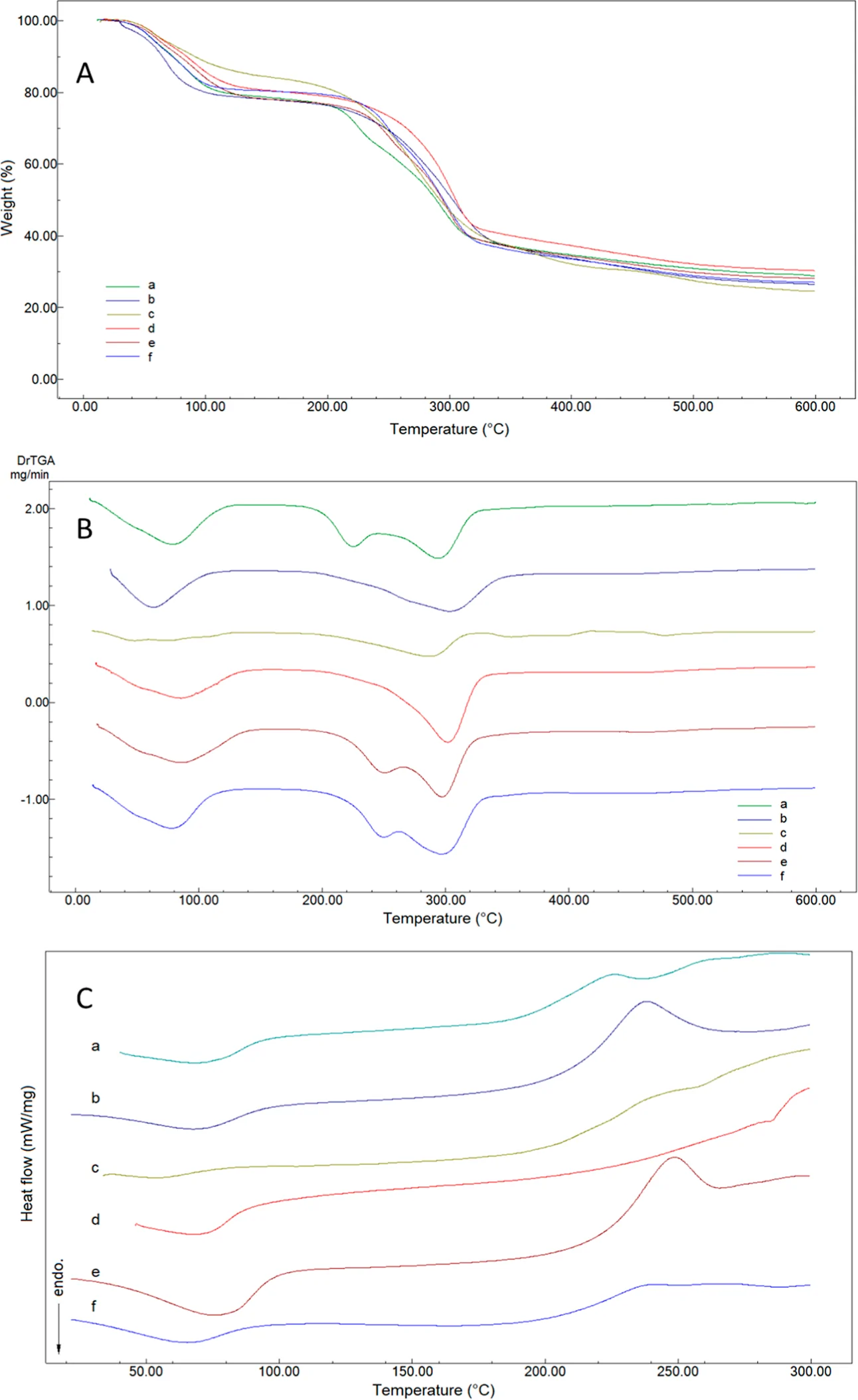
TGA thermograms (A), Dr-TGA thermograms (B), and DSC curves (C) of BC samples obtained at different incubation times: (a) second day, (b) third day, (c) fourth day, (d) fifth day, (e) sixth day, and (f) seventh day.

Adequate flexibility is also a desirable property for the BC. Therefore, DSC thermograms were also recorded to determine the thermal stability of the BCs. The DSC results are given in Figure 3C. DSC analysis (Figure 3C) of the resulting BC structures revealed a clear *T*_g_ transition, particularly at lower temperatures. It was determined that *T*_g_ temperatures were approximately 20 °C, and as the day progressed, the *T*_g_ temperature also increased due to increased stacking density and H-bonding. The *T*_g_ temperatures of the BCs obtained from day 2 to day 7 were determined as 16.3, 19.8, 20.5, 22.2, 24.3, and 25.1 °C, respectively. Notably, all values were below body temperature and close to ambient room temperature, indicating that BC structures are suitable for use as membranes. The results suggest that the thermal properties of the BCs are suitable for membrane applications (Figure 3). The morphology of the BC fibers, including parameters such as fiber diameter, porosity, and pore distribution, is crucial for application.

SEM analyses from day 2 to day 7 are presented comparatively in Figure 4 at both low (40000×) and high (80000×) magnifications. When the low-magnification SEM structures were examined, it was observed that on day 2, the structure exhibits a smooth, paperlike morphology. In these structures, overlapping fiber networks are clearly visible, creating microvoids where they intersect. Overall, the structure appears to be homogeneous and uniform without detectable impurities. Furthermore, the pores were distributed evenly, and the morphology resembled a polymeric network. By day 7, an extremely tightly packed polymeric network was observed. Because of increased agglomeration in the polymeric network, the distance between the pores had decreased significantly, which may lead to a reduction in membrane permeability.

**Figure 4:**
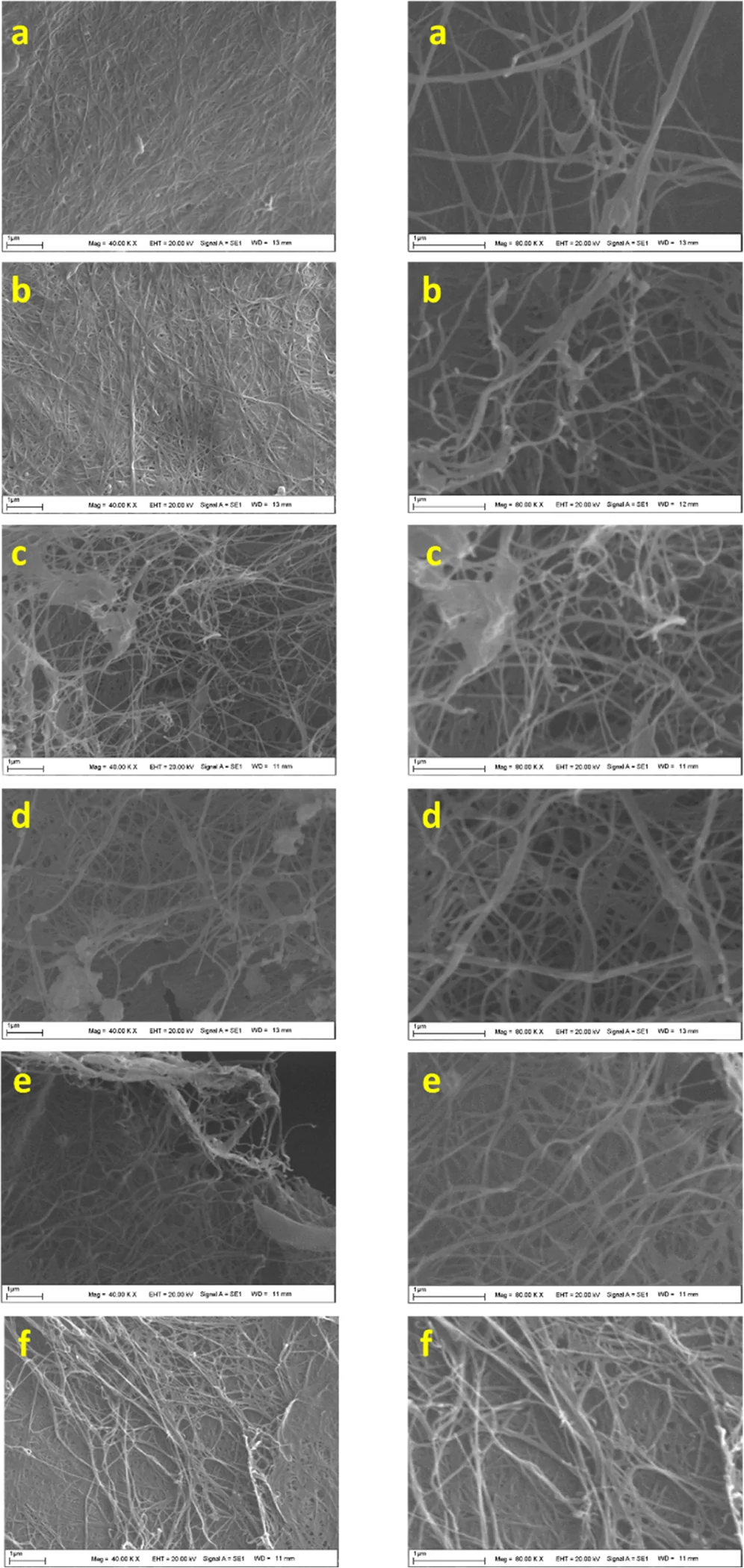
SEM images of BC samples obtained at different incubation times: (a) second day, (b) third day, (c) fourth day, (d) fifth day, (e) sixth day, and (f) seventh day.

To examine the relevant fiber diameters in more detail, SEM measurements were performed at 100 000× magnification, enabling precise visualization of fiber dimensions and structures. Average fiber diameters were determined for each day, and the corresponding fiber diameter distribution graphs are plotted (Figure 5). On the basis of the fiber diameter distribution ranges, the optimal fiber structure for the membrane was also determined in the BC obtained on the third day.

**Figure 5:**
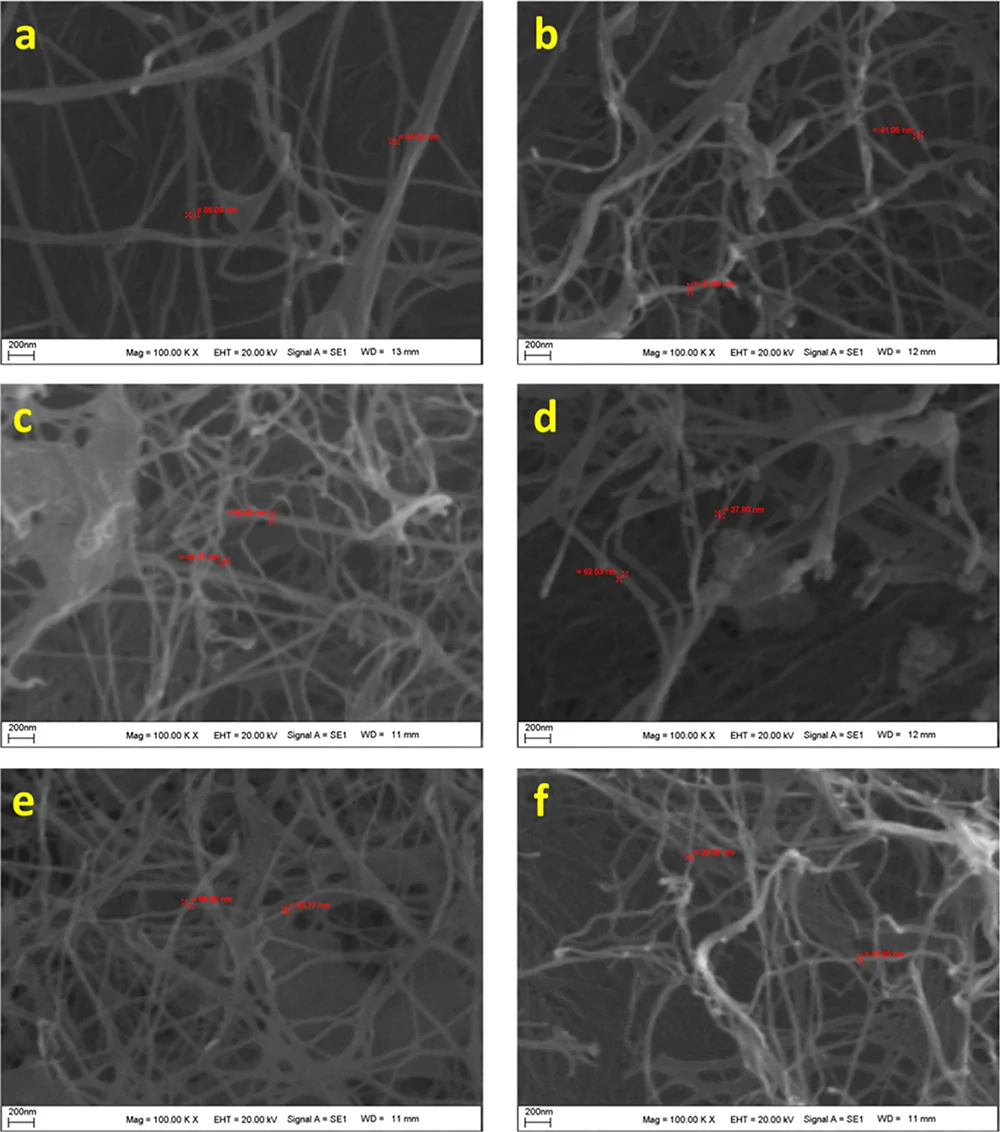
Fiber measurement images of BC samples obtained at different incubation times: (a) second day, (b) third day, (c) fourth day, (d) fifth day, (e) sixth day, and (f) seventh day.

The basic SEM images in Figure 5 were evaluated for each structure, and fiber diameters were examined. The fiber diameters of the BCs ranged from approximately 21 and 66 nm across different days of incubation.^6^ Membrane thickness measurements of BCs are given in Figure 6. The thickness increased progressively over time. The membrane thickness on the second day was 2276 μm, on the third day, it was 3400 μm, on the fourth day, it was 3575 μm, on the fifth day, it was 3940 μm, on the sixth day, it was 4270 μm, and on the seventh day, it was 5038 μm. Similarly, wet weight measurements of the BCs indicated an incubation time dependent increase. The BC obtained on day 3 had a wet weight of approximately 5 g, whereas the BC on day 7 weighed about 20 g.

**Figure 6:**
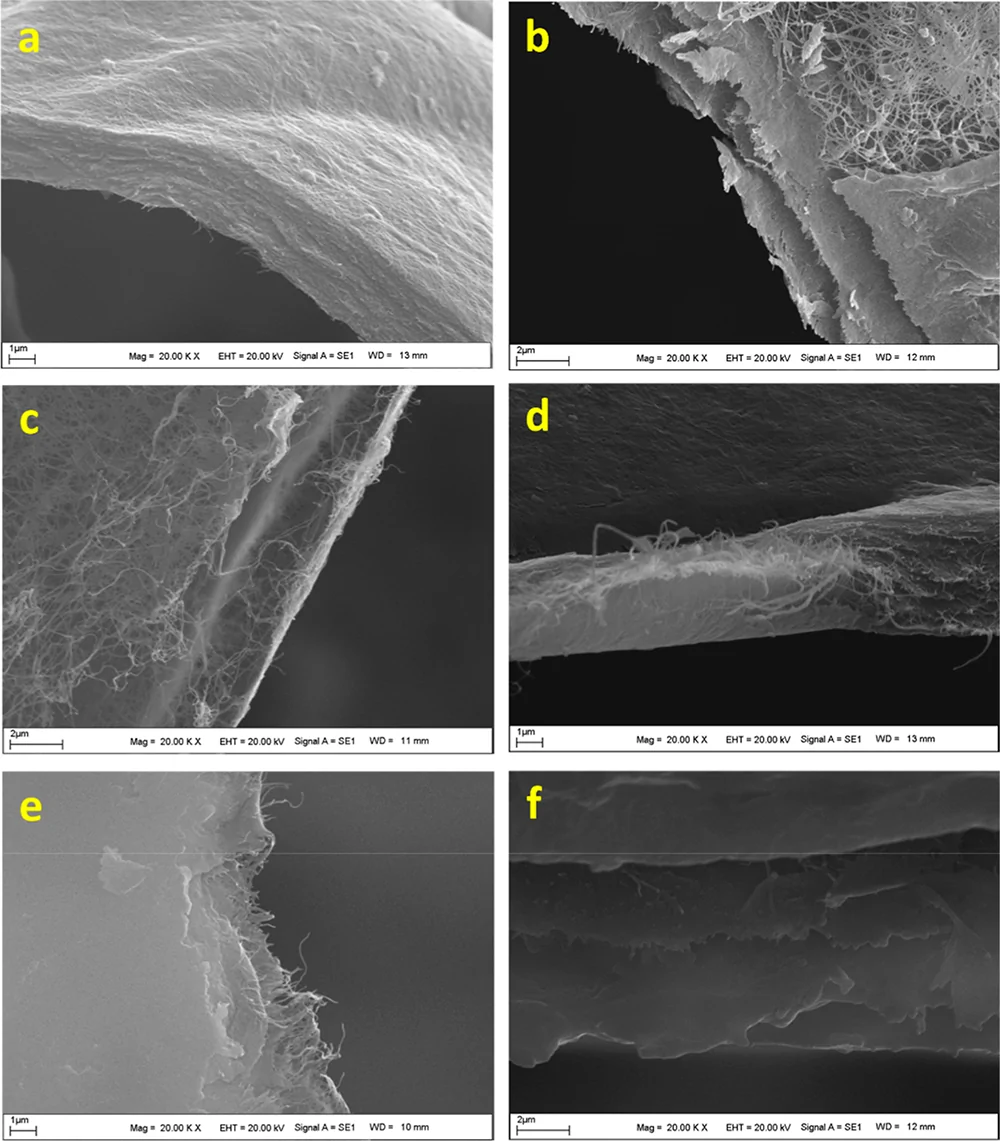
Vertical section SEM images of BC fiber structures obtained at different incubation times: (a) second day, (b) third day, (c) fourth day, (d) fifth day, (e) sixth day, and (f) seventh day.

The surface topography was created to determine the membrane structure using ImageJ (Figure 7). The results showed that a distinct and continuous membrane structure began to form on day 3, with a gradual thickening observed at longer incubation times. ImageJ analysis revealed that although a clear fiber structure was formed as early as day 2, an ideal fiber structure was achieved on day 3.

**Figure 7:**
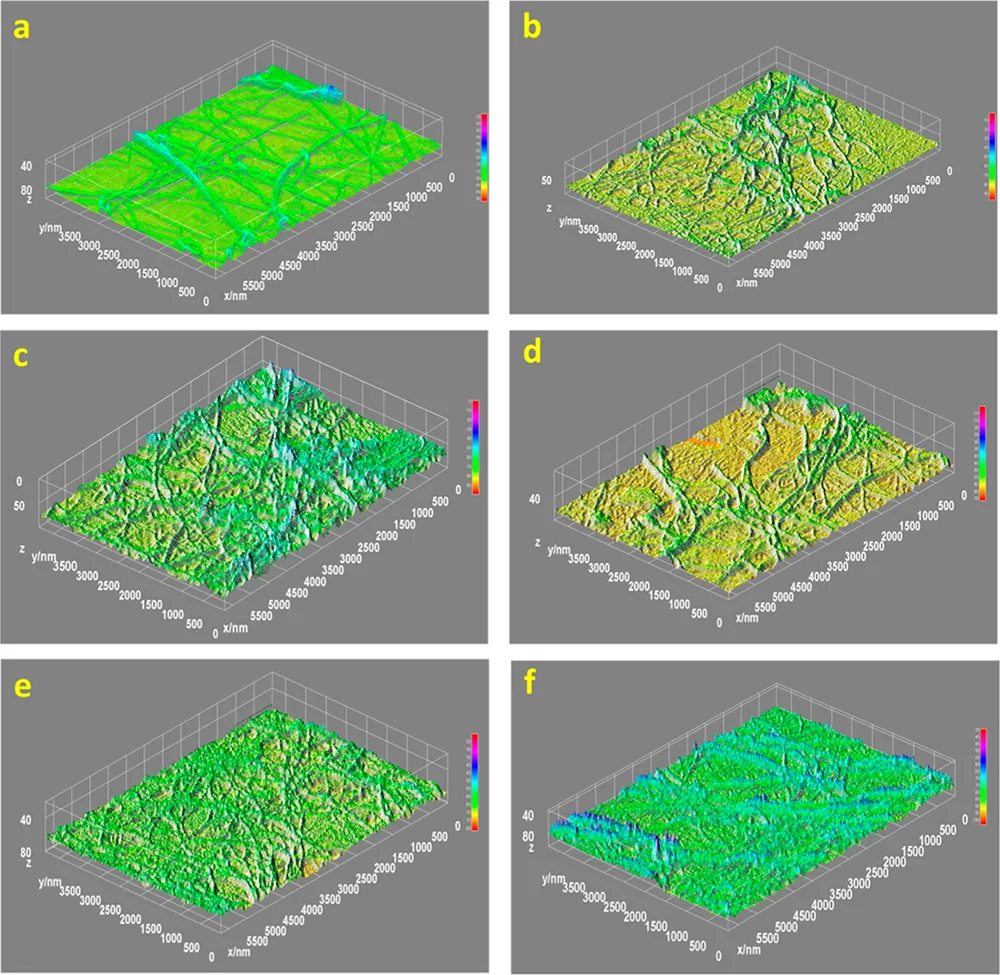
Surface topography images of BC structures obtained at different incubation times: (a) second day, (b) third day, (c) fourth day, (d) fifth day, (e) sixth day, and (f) seventh day.

However, at later incubation stages, the membrane structure was observed to narrow, leading to the reduction in membrane permeability, which is undesirable for membrane filtration processes. Therefore, it is critical to determine the optimum fiber structure and thickness. SEM analyses conducted in this study clearly demonstrated that the membrane structure obtained on day 3 was in an ideal form.

All membranes consist of cellulosic units and thus exhibit intrinsic polarity. However, because surface roughness and membrane distribution vary, liquid contact angle measurements were performed (Figure 8). The measured contact angles increased over time: 38.27°, 40.89°, 44.86°, 50.74°, 62.06°, and 69.15° on days 2 through 7, respectively. Since the main chemical structure is the same, this increase is attributed to changes in surface roughness. These results suggest the membranes become smoother over time, with the surface roughness decreasing toward day 7.

**Figure 8:**
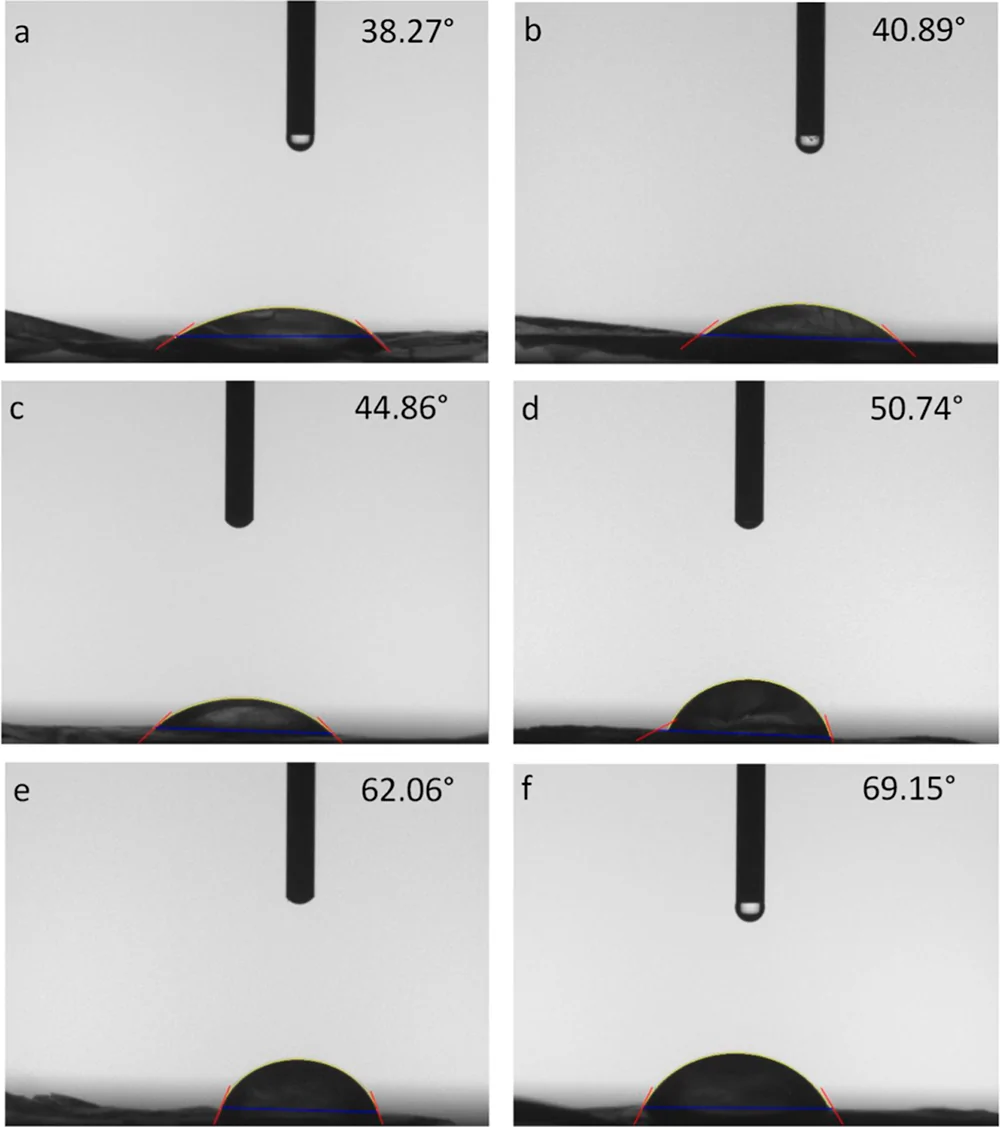
Liquid contact angle measurement images of BC fiber structures obtained at different incubation times: (a) second day, (b) third day, (c) fourth day, (d) fifth day, (e) sixth day, and (f) seventh day.

The membranes prepared for this study are expected to exhibit distinct mechanical properties. In filtration applications, membranes are subjected to significant stress due to both vacuum pressure and the mechanical forces from the liquid flow. To assess their suitability for practical use and resistance to these stresses, these mechanical effects, mechanical analyses were performed on each sample. The mechanical test results for dry and wet conditions are given in Figure 9, Table 3, Figure 10, and Table 4.

**Figure 9:**
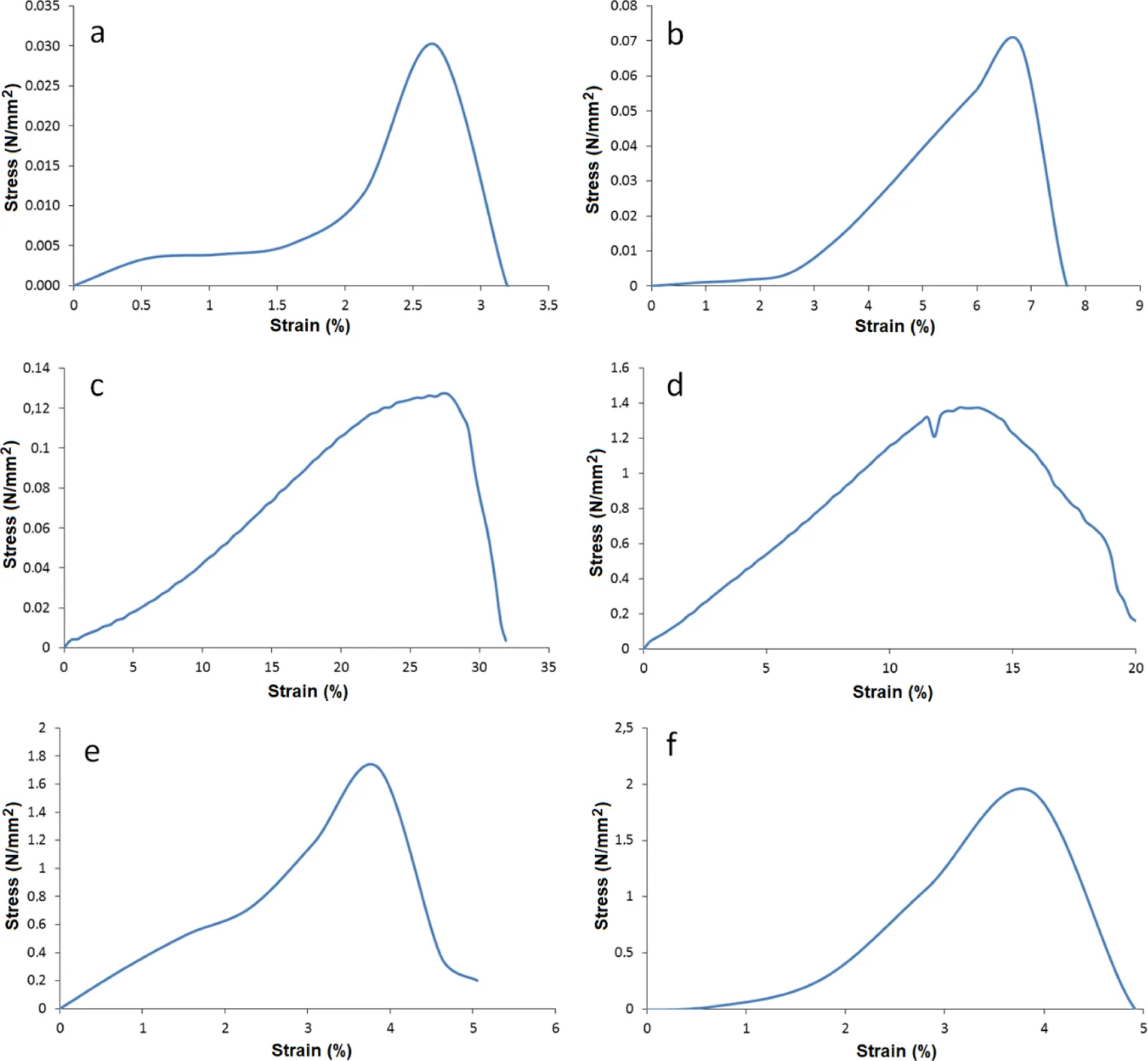
Mechanical analysis test results of dry BC fiber structures obtained at different incubation times: (a) second day, (b) third day, (c) fourth day, (d) fifth day, (e) sixth day, and (f) seventh day.

**Table 3:** Mechanical Analysis Test Results of Dry BC Fiber Structures Obtained at Different Incubation Times: (a) Second Day, (b) Third Day, (c) Fourth Day, (d) Fifth Day, (e) Sixth Day, and (f) Seventh Day

incubation time (day)	ultimate tensile strength (N/mm^2^)	peak load (N)	elastic modulus (kN/mm^2^)	% fracture strain (mm/mm)
2	0.030	0.352	0.005	2.666
3	0.068	0.879	0.016	6.803
4	0.127	1.641	0.017	27.308
5	1.355	11.013	0.026	13.354
6	1.720	20.210	0.028	3.850
7	1.934	29.011	0.039	3.800

**Figure 10:**
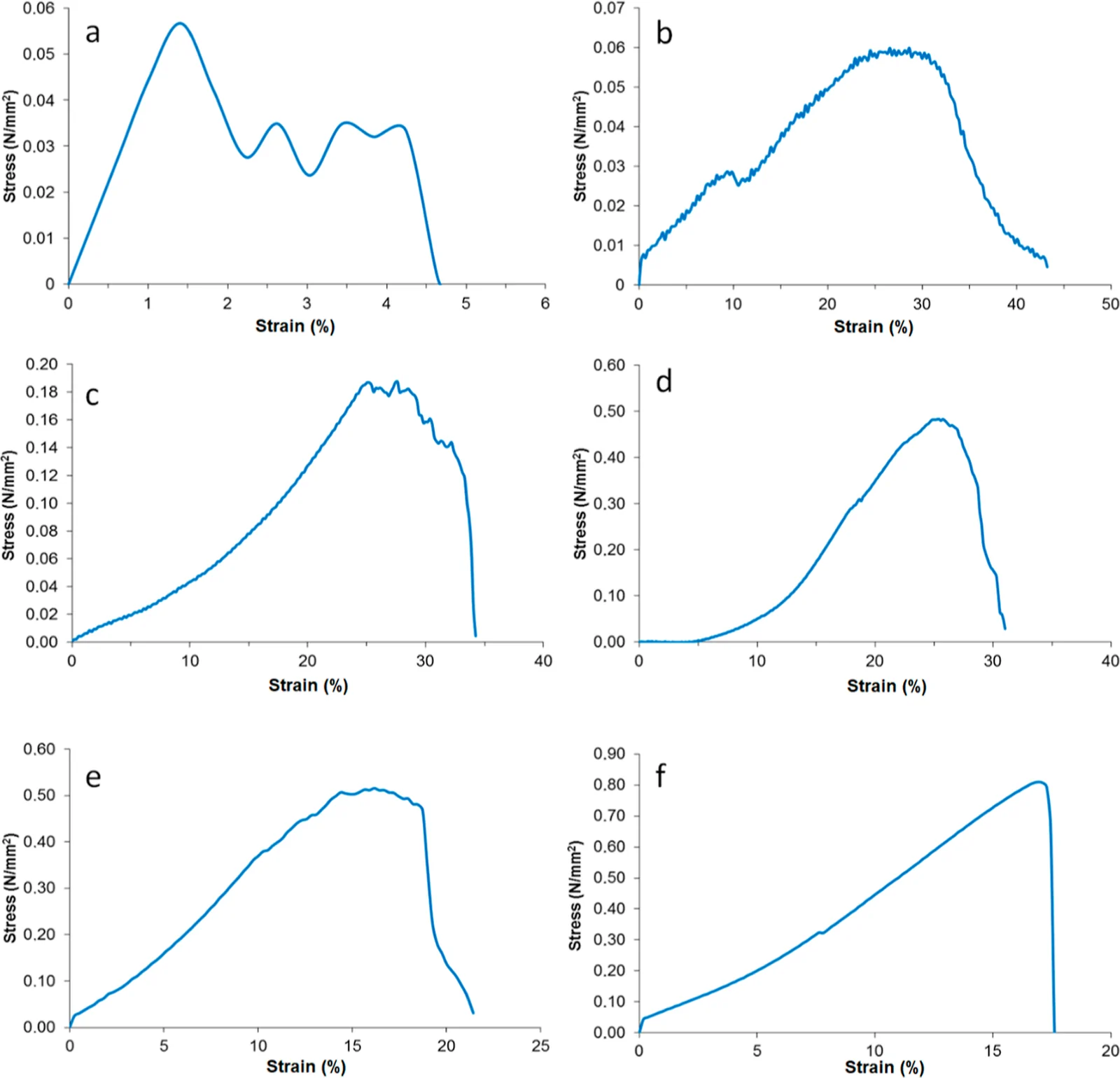
Mechanical analysis test results of wet BC fiber structures obtained at different incubation times: (a) second day, (b) third day, (c) fourth day, (d) fifth day, (e) sixth day, and (f) seventh day.

**Table 4:** Mechanical Analysis Test Results of Wet BC Fiber Structures Obtained at Different Incubation Times: (a) Second Day, (b) Third Day, (c) Fourth Day, (d) Fifth Day, (e) Sixth Day, and (f) Seventh Day

incubation time (day)	ultimate tensile strength (N/mm^2^)	peak load (N)	elastic modulus (kN/mm^2^)	% fracture strain (mm/mm)
2	0.056	1.11	0.007	1.415
3	0.059	1.13	0.003	26.091
4	0.182	5.46	0.012	25.956
5	0.482	6.30	0.015	25.093
6	0.515	10.89	0.019	16.172
7	0.808	43.61	0.006	16.065

### 3.2 Use of Bacterial Cellulose as Bacterial Membrane Filters

Due to superior physicochemical and mechanical properties of BC, various studies are being conducted on its use as membrane filters in various applications.^30–32^ The purified BC membranes obtained on day 3 were evaluated for their ability to filter *E. coli* and *S. aureus*. Bacterial suspensions were prepared separately at known concentrations and filtered through sterilized BC membranes under aseptic conditions. Viable cell counts were performed before and after filtration. No bacterial growth was detected in the filtrates following filtration, indicating complete bacterial retention (Figure 11). These results demonstrate that BC can be used as a membrane filter for microorganism removal, consistent with previous findings.^6^ The reusability of BC membranes was also assessed by repeating the filtration process five consecutive times. In all filtration cycles, including the fifth repetition, bacterial passage was completely prevented, confirming the durability and reusability of BC membranes. It was reported that when *Komagataeibacter nataicola* TISTR was cultivated in molasses media for 10 days, it produced BC with effective *E. coli* removal activity from suspension.^19^ The lyophilized (dried) bacterial cellulose membrane was also used as a membrane for filtration of bacteria. It was initially immersed in distilled water for 24 h and then, the bacterial suspension was filtered through this membrane. The absence of bacterial cells in the filtrate following filtration demonstrated that the dried membrane is also suitable to be used as a membrane filter.

**Figure 11:**
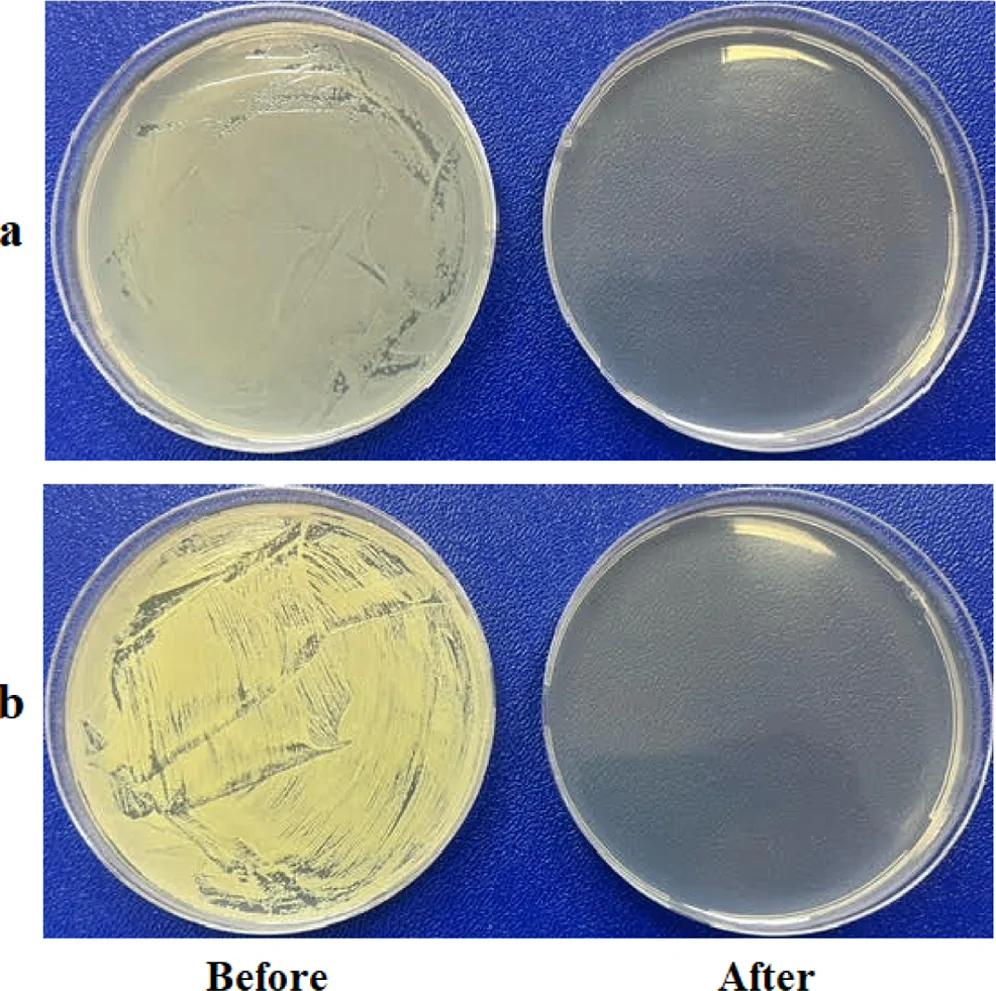
Images of *E. coli* (a) and *S. aureus* (b) cultures incubated on Mueller–Hinton agar plates before and after filtration through BC membranes.

### 3.3 Membrane Surface Images of Bacterial Cellulose Membranes after the Filtration Process

Analysis indicated that the BC produced on day 3 showed the most favorable membrane structure. Therefore, this membrane was selected for use as a filtration membrane and achieved a 100% reduction in bacterial load. To confirm the retention of bacterial cells on the membrane surface following filtration, SEM (Figures 12 and 13) and EDX analyses (Figure 14) were performed.

**Figure 12:**
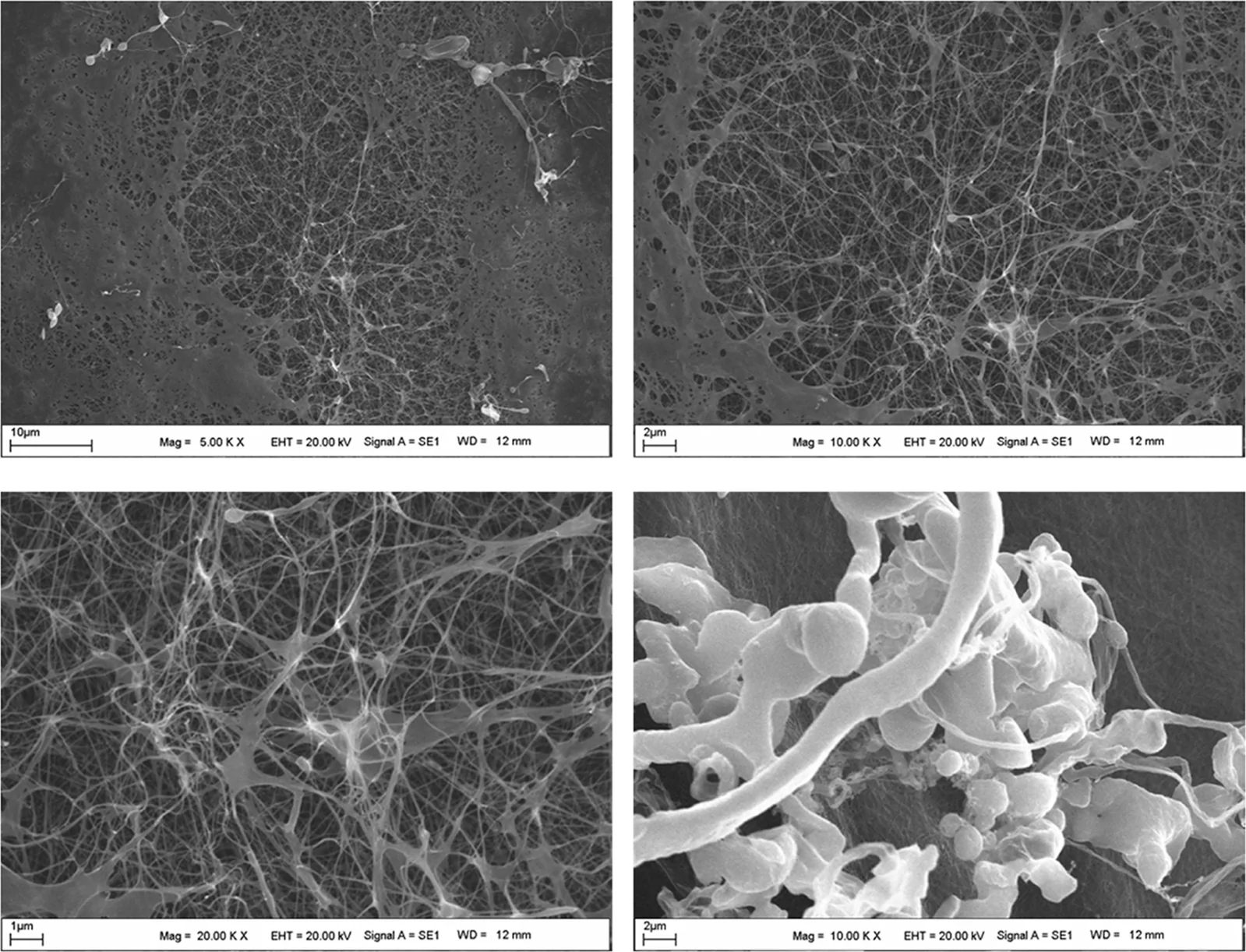
BC membrane surface images after filtration of *E. coli*.

**Figure 13:**
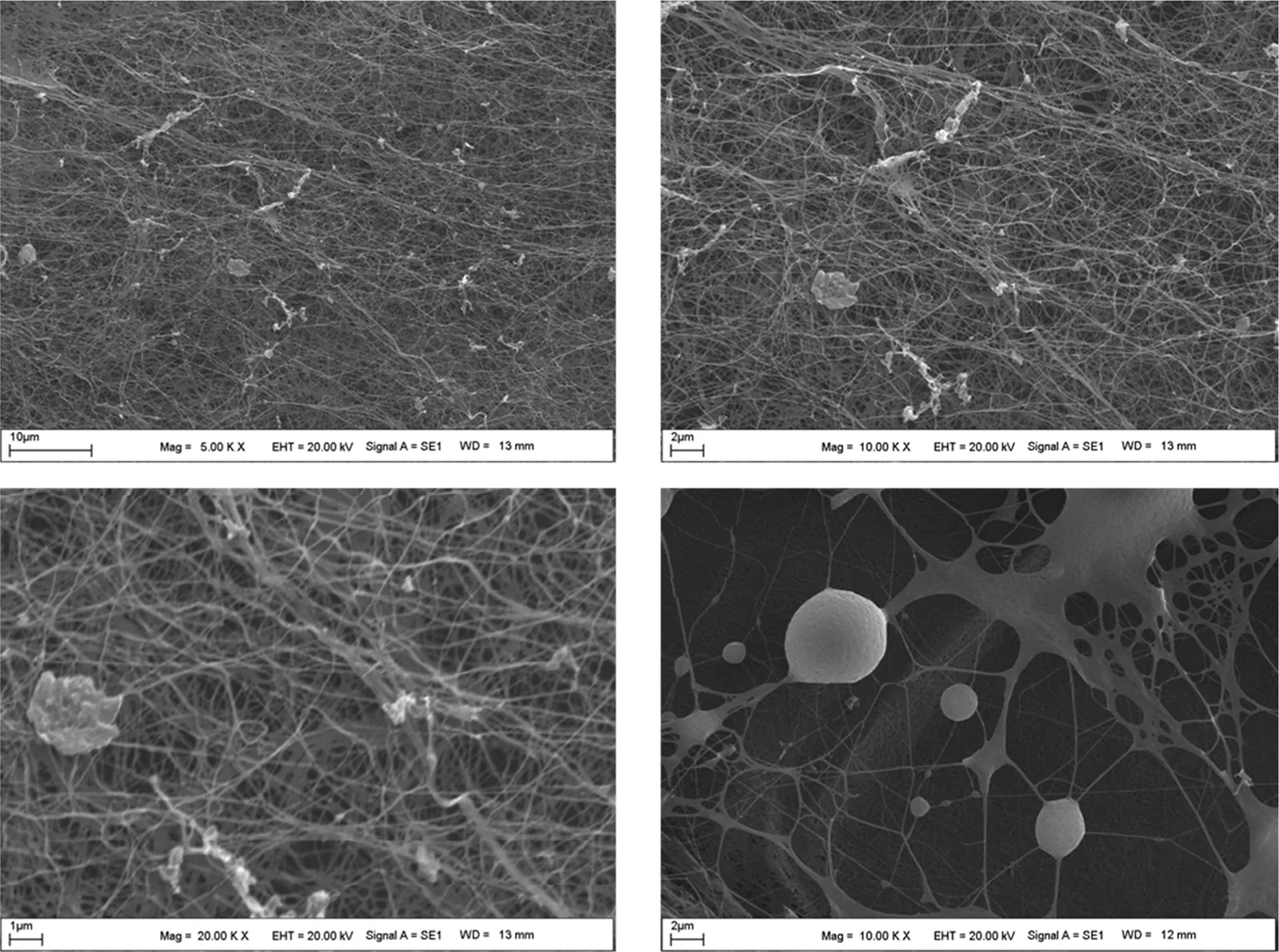
BC membrane surface images after filtration of *S. aureus*.

**Figure 14:**
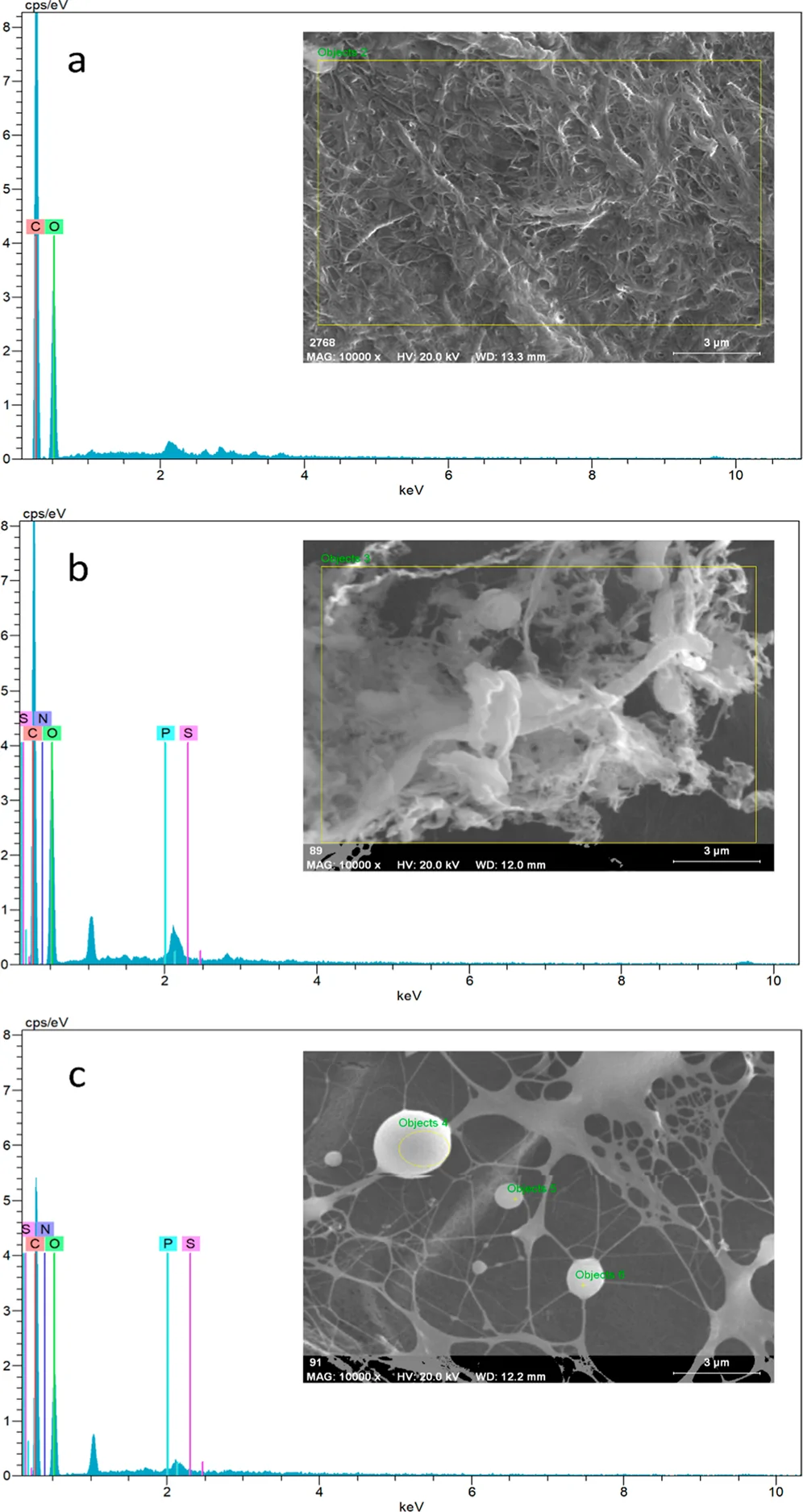
EDX spectra of pure BC membrane (a), *E. coli*-filtered membrane (b), and *S. aureus*-filtered membrane (c).

At higher magnifications, *E. coli* cells were observed clustering on the membrane surface (Figure 12). In certain areas, bacteria appeared to accumulate, and attachments between the fiber structures become more pronounced. At very high magnifications, *E. coli* cells can be clearly observed entrapped within the membrane fiber network. Figure 13 also shows the presence of *S. aureus* on the membrane surface after filtration. Spherical bacterial cells were detected between the membrane fibers, indicating highly effective bacterial retention. No structural damage or morphological deterioration of the membrane was observed after the filtration process, supporting the membrane’s potential for repeated use.

The elemental composition of the membrane surface was analyzed before and after the bacterial filtration. In contrast, the pure membrane (membrane before filtration) showed additional elements corresponding to nitrogen, phosphorus, and sulfur on membranes after filtration of *E. coli* and *S. aureus*. The EDX spectrum of the pure membrane showed only carbon and hydrogen peaks, consistent with the cellulose structure. However, membranes exposed to bacterial filtration exhibited additional peaks, such as nitrogen, phosphorus, and sulfur, confirming the presence of bacteria on the membrane surface (Figure 14).

Before EDX spectra were obtained, the sample surfaces were coated with Au/Pd to ensure conductivity. Due to these coatings, an Au Mα peak of 2.12 keV and a Pd Lα peak of 2.84 keV were observed in all three samples. However, membranes exposed to bacterial filtration exhibited additional peaks of N Kα at 0.39 keV, P at 2.01 keV, and S Kα at 2.31 keV. Nitrogen, phosphorus, and sulfur confirm the presence of adherent bacteria on the membrane surface. Comparing the EDX spectra of pure BC membranes with those of BC membranes after filtration proves that an ideal filtration process was achieved.

To confirm that the observed elemental peaks originated from microbial cells, surface elemental mapping of *E. coli*- and *S. aureus*-filtered membranes was performed (Figures 15 and 16). Carbon, oxygen, phosphorus, and sulfur were clearly detected on the membrane surface. This result also indicates effective bacterial retention by the BC membrane during filtration.

**Figure 15:**
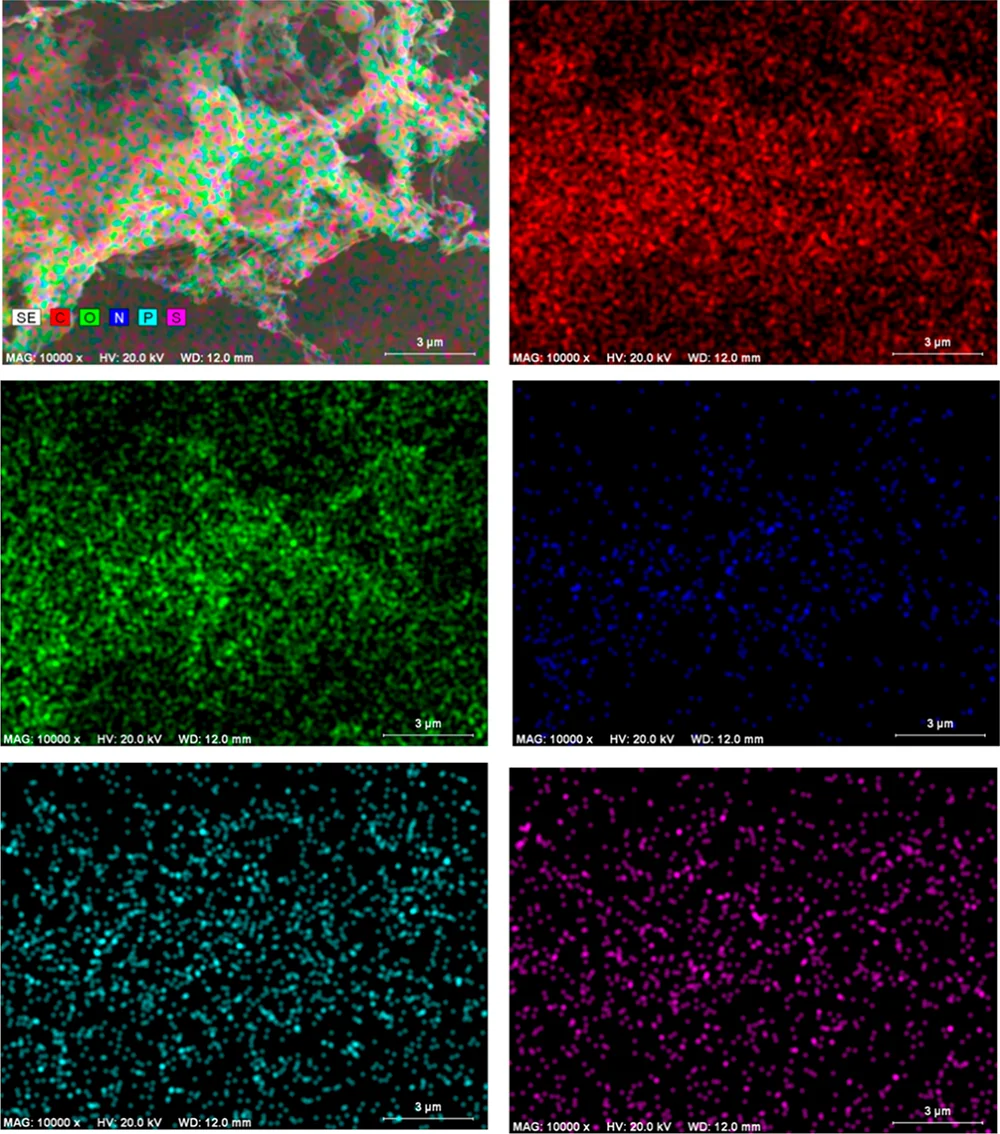
Elemental maps of the BC membrane surface after filtration of *E. coli*.

**Figure 16:**
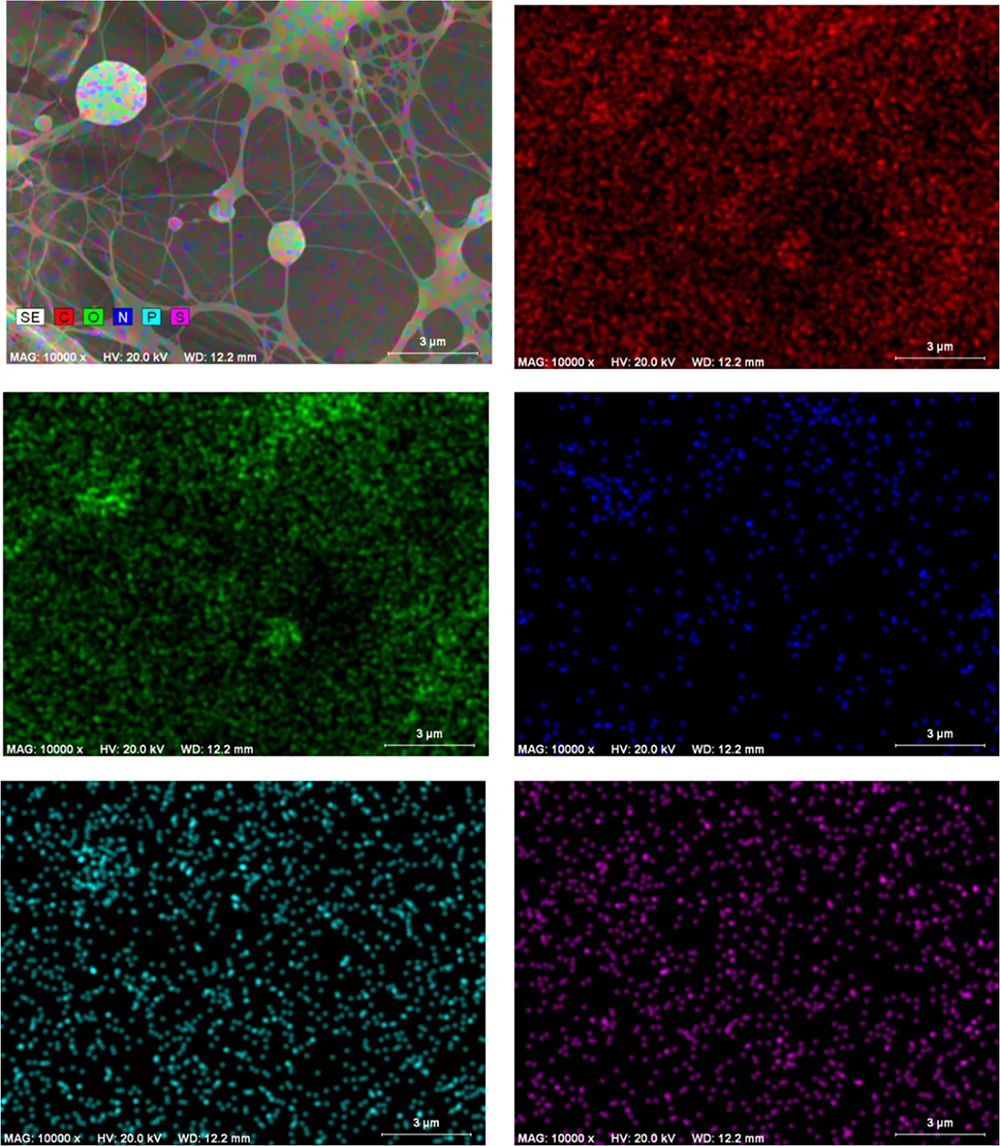
Elemental maps of the BC membrane surface after filtration of *S. aureus*.

The usability of BC for the filtration of textile dye Astrazon Red dye and model dye Methylene Blue, as well as waste oil/water mixture was also investigated. The results showed that while Astrazon Red dye was decolorized by %46, Methylene Blue exhibited %76 decolorization after filtration. For filtration of waste oil/water mixture, a mixture consisting of 10 mL waste oil and 10 mL water was prepared and filtered through BC^33^ (Figure 17). Since the resulting BC membrane is a hydrophilic membrane, it filtered water. While the viscosity of the waste oil/water mixture was 98.36 ± 8.18, the viscosity of the filtrate was measured as 33.42 ± 7.97 after filtration. The results demonstrated that the BC membrane may also serve as an effective biologic material for filtration of these types of pollutants.

**Figure 17:**
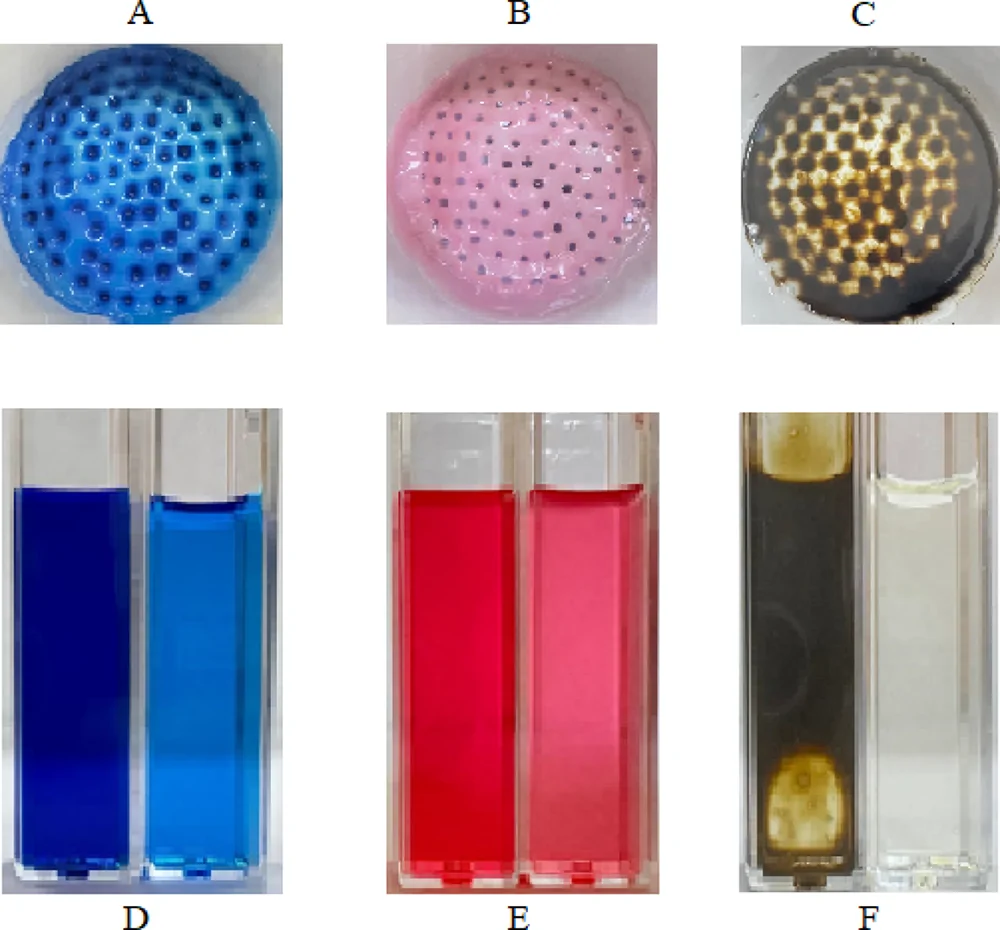
Photographs of BC membranes and dyes and oil/water mixture solutions after filtration. BC membranes after filtration of Methylene Blue dye (A), Astrazon Red dye (B), and oil/water mixture (C). Color of Methylene Blue (D), Astrazon Red (E), and waste oil/water mixture (F) solutions before (left) and after (right) filtration through the BC membrane.

FTIR, thermal, and morphological analyses of BC showed that it possesses an ideal membrane structure. In particular, FTIR and X-ray spectrum analyses proved that the produced BC structure is pure and very clean, with no peaks caused by impurities. The high crystallinity obtained in X-ray analyses indicates that the obtained membrane structure will be stable. This structural stability was also confirmed by mechanical analyses, showing that it possesses the mechanical strength required for filtration processes. Furthermore, the thermal stability of the produced BC structure up to approximately 230 °C in thermal analyses indicates that it can be sterilized and used in filtration processes above room temperature. Morphological analyses performed on the BC structure proved that an ideal pore and surface structure was obtained. In conclusion, the characterization and filtration results confirmed that the BC structure produced with *Gluconacetobacter xylinus* *NRRL B-759* exhibits ideal membrane properties.

## 4 Conclusion

In this study, a comprehensive series of characterization and filtration experiments were conducted. Detailed analyses of the membrane were performed over a period from the second to the seventh day, including elemental analysis, FTIR, XRD, TGA, thermal stability assessment, SEM, liquid contact angle measurements, and mechanical testing. The membrane’s effectiveness in bacterial filtration was subsequently demonstrated and further confirmed through SEM and energy-dispersive X-ray (EDX) analyses, as well as surface elemental mapping of the filtered membrane. The primary objective of this study was to produce pure BC using *G. xylinus* and to evaluate its suitability for use as membrane filter. The production of high-purity bacterial cellulose (BC) using *G. xylinus* was successfully demonstrated, and its suitability as a membrane filter was evaluated. BC formation strongly depended on incubation time. Among the tested BC samples, the membrane produced on day 3 showed the most favorable structural organization, crystallinity, and mechanical performance. Thermal analysis confirmed the sufficient stability. Tensile testing revealed adequate strength in both wet and dry conditions. Membrane filtration experiments showed complete bacterial retention with no structural deterioration and excellent reusability over multiple cycles. SEM and EDX analyses and surface elemental mapping confirmed this bacterial retention. The findings indicate that the BC membranes, particularly those produced on day 3, have significant potential for microorganism filtration. Instead of producing bacteria for a long time to obtain an effective membrane for bacterial filtration, it would be sufficient to produce the BC membrane in the shortest incubation time and obtain an environmentally friendly membrane. BC membrane may also serve as an effective biologic material for filtration of various pollutants.

## Data Availability

There is no legal/ethical problem in publishing the data.
